# Pyridine-3-carbonitrile–chloranilic acid–acetonitrile (2/1/2)

**DOI:** 10.1107/S1600536809036605

**Published:** 2009-09-16

**Authors:** Kazuma Gotoh, Hiroyuki Ishida

**Affiliations:** a Department of Chemistry, Faculty of Science, Okayama University, Okayama 700-8530, Japan

## Abstract

In the crystal structure of the title compound, 2C_6_H_4_N_2_·C_6_H_2_Cl_2_O_4_·2C_2_H_3_N, the two symmetry-related pyridine-3-carbonitrile mol­ecules are linked to either side of a chloranilic acid (systematic name: 2,5-dichloro-3,6-dihydr­oxy-1,4-benzoquinone) mol­ecule *via* inter­molecular O—H⋯N hydrogen bonds, giving a centrosymmetric 2:1 unit. The dihedral angle between the pyridine ring and the chloranilic acid plane is 26.71 (6)°. In addition, the two acetonitrile mol­ecules are linked to either side of the 2:1 unit through C—H⋯N hydrogen bonds, forming a 2:1:2 aggregate. These 2:1:2 aggregates are further linked by weak inter­molecular C—H⋯N and C—H⋯O hydrogen bonds, forming a tape along the *c* axis.

## Related literature

For related structures, see, for example: Gotoh *et al.* (2009 ▶); Gotoh, Asaji & Ishida (2008 ▶); Gotoh, Nagoshi & Ishida (2008 ▶).
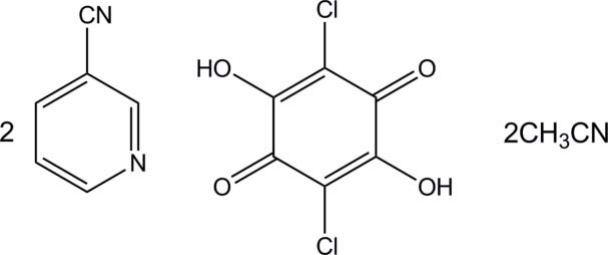


## Experimental

### 

#### Crystal data

2C_6_H_4_N_2_·C_6_H_2_Cl_2_O_4_·2C_2_H_3_N
*M* *_r_* = 499.31
Triclinic, 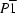

*a* = 3.91269 (16) Å
*b* = 10.8937 (9) Å
*c* = 13.5966 (5) Å
α = 105.302 (4)°
β = 90.0058 (14)°
γ = 90.847 (5)°
*V* = 558.93 (6) Å^3^
*Z* = 1
Mo *K*α radiation
μ = 0.33 mm^−1^
*T* = 180 K
0.32 × 0.25 × 0.15 mm

#### Data collection

Rigaku RAXIS-RAPID II diffractometer
Absorption correction: numerical (**ABSCOR**; Higashi, 1995 ▶) *T*_min_ = 0.906, *T*_max_ = 0.951
7721 measured reflections
3232 independent reflections
2592 reflections with *I* > 2σ(*I*)
*R*_int_ = 0.031

#### Refinement

*R*[*F* ^2^ > 2σ(*F* ^2^)] = 0.035
*wR*(*F* ^2^) = 0.099
*S* = 1.07
3232 reflections
159 parameters
H atoms treated by a mixture of independent and constrained refinement
Δρ_max_ = 0.44 e Å^−3^
Δρ_min_ = −0.28 e Å^−3^

### 

Data collection: *PROCESS-AUTO* (Rigaku/MSC, 2004 ▶); cell refinement: *PROCESS-AUTO*; data reduction: *CrystalStructure* (Rigaku/MSC, 2004 ▶); program(s) used to solve structure: *SHELXS97* (Sheldrick, 2008 ▶); program(s) used to refine structure: *SHELXL97* (Sheldrick, 2008 ▶); molecular graphics: *ORTEP-3* (Farrugia, 1997 ▶); software used to prepare material for publication: *CrystalStructure* and *PLATON* (Spek, 2009 ▶).

## Supplementary Material

Crystal structure: contains datablocks General, I. DOI: 10.1107/S1600536809036605/lh2901sup1.cif

Structure factors: contains datablocks I. DOI: 10.1107/S1600536809036605/lh2901Isup2.hkl

Additional supplementary materials:  crystallographic information; 3D view; checkCIF report

## Figures and Tables

**Table 1:** Hydrogen-bond geometry (Å, °)

*D*—H⋯*A*	*D*—H	H⋯*A*	*D*⋯*A*	*D*—H⋯*A*
O2—H2⋯N1	0.92 (3)	1.75 (3)	2.6111 (17)	154 (3)
O2—H2⋯O1^i^	0.92 (3)	2.25 (3)	2.6824 (14)	108 (2)
C4—H4⋯N2^ii^	0.95	2.46	3.292 (2)	146
C6—H6⋯N3	0.95	2.57	3.385 (2)	144
C7—H7⋯O1^iii^	0.95	2.48	3.4248 (18)	172
C11—H11*A*⋯N2	0.98	2.62	3.341 (2)	130

Symmetry codes: (i) 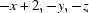; (ii) 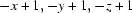; (iii) 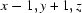.

**Fig. 1.**
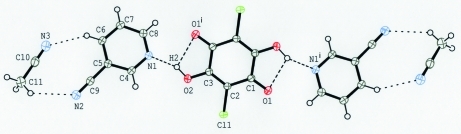
The molecular structure of the title compound, with the atom-labeling. Displacement ellipsoids of non-H atoms are drawn at the 50% probability level. The dashed lines indicate O—H···O, O—H···N and C—H···N hydrogen bonds. [Symmetry code: (i) -x + 2, -y, -z].

**Fig. 2.**
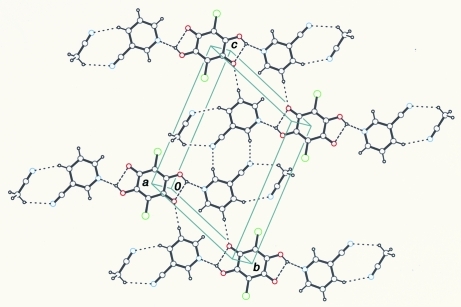
A packing diagram of (I), showing a molecular tape running along the c axis. The dashed lines indicate O—H···O, O—H···N, C—H···N and C—H···O hydrogen bonds.

## supplementary crystallographic information

### Comment

The title compound, (I), was prepared in order to extend our study on *D*—H···*A* hydrogen bonding (*D* = N, O, or C; *A* = N, O or Cl) in amine–chloranilic acid systems (Gotoh, Asaji & Ishida, 2008; Gotoh *et al.*, 2009).

In the crystal structure of the title compound, two pyridine-3-carbonitrile molecules, one chloranilic acid molecule and two acetonitrile molecules are linked by O—H···N and C—H···N hydrogen bonds (Table 1) to afford a 2:1:2 aggregate (Fig. 1). The O···N distance [2.6111 (17) Å] between the acid and the base is comparable to that of 2.610 (3) Å in pyridine-4-carbonitrile–chloranilic acid (1/1), where the H atom in the O···H···N hydrogen bond is disordered (Gotoh, Nagoshi & Ishida, 2008), but in the title compound no distinct evidence of H disorder was observed in a difference Fourier map. The 2:1:2 aggregates are linked by weak intermolecular C—H···N and C—H···O hydrogen bonds, forming a tape along the *c* axis (Fig. 2). A short contact between the adjacent C≡N bonds of acetonitrile molecules is observed [C10···C10^iii^ 3.314 Å; symmetry code: (iii) -*x* - 1, -*y* + 2, -*z* + 1].

### Experimental

Single crystals were obtained by slow evaporation from an acetonitrile solution (120 ml) of chloranilic acid (250 mg) and pyridine-3-carbonitrile (250 mg) at room temperature.

### Refinement

C-bound H atoms were positioned geometrically (C—H = 0.95 or 0.98 Å) and refined as riding, allowing for free rotation of the methyl group. *U*_iso_(H) values were set at 1.2*U*_eq_(C) or 1.5*U*_eq_(methyl C). The O-bound H atom was found in a difference Fourier map and refined isotropically. The refined O—H distance is 0.92 (3) Å.

### Crystal data

**Table:**

2C_6_H_4_N_2_·C_6_H_2_Cl_2_O_4_·2C_2_H_3_N	*Z* = 1
*M**_r_* = 499.31	*F*(000) = 256.00
Triclinic, *P*1	*D*_x_ = 1.483 Mg m^−^^3^
Hall symbol: -P 1	Mo *K*α radiation, λ = 0.71075 Å
*a* = 3.91269 (16) Å	Cell parameters from 6412 reflections
*b* = 10.8937 (9) Å	θ = 3.1–30.1°
*c* = 13.5966 (5) Å	µ = 0.33 mm^−^^1^
α = 105.302 (4)°	*T* = 180 K
β = 90.0058 (14)°	Block, brown
γ = 90.847 (5)°	0.32 × 0.25 × 0.15 mm
*V* = 558.93 (6) Å^3^	

### Data collection

**Table:**

Rigaku RAXIS-RAPID II diffractometer	2592 reflections with *I* > 2σ(*I*)
Detector resolution: 10.00 pixels mm^-1^	*R*_int_ = 0.031
ω scans	θ_max_ = 30.0°
Absorption correction: numerical (*ABSCOR*; Higashi, 1995)	*h* = −5→5
*T*_min_ = 0.906, *T*_max_ = 0.951	*k* = −15→15
7721 measured reflections	*l* = −19→17
3232 independent reflections	

### Refinement

**Table:**

Refinement on *F*^2^	Primary atom site location: structure-invariant direct methods
Least-squares matrix: full	Secondary atom site location: difference Fourier map
*R*[*F*^2^ > 2σ(*F*^2^)] = 0.035	Hydrogen site location: inferred from neighbouring sites
*wR*(*F*^2^) = 0.099	H atoms treated by a mixture of independent and constrained refinement
*S* = 1.07	*w* = 1/[σ^2^(*F*_o_^2^) + (0.0495*P*)^2^ + 0.1506*P*] where *P* = (*F*_o_^2^ + 2*F*_c_^2^)/3
3232 reflections	(Δ/σ)_max_ < 0.001
159 parameters	Δρ_max_ = 0.44 e Å^−^^3^
0 restraints	Δρ_min_ = −0.28 e Å^−^^3^

### Special details

**Table:**

Geometry. All e.s.d.'s (except the e.s.d. in the dihedral angle between two l.s. planes) are estimated using the full covariance matrix. The cell e.s.d.'s are taken into account individually in the estimation of e.s.d.'s in distances, angles and torsion angles; correlations between e.s.d.'s in cell parameters are only used when they are defined by crystal symmetry. An approximate (isotropic) treatment of cell e.s.d.'s is used for estimating e.s.d.'s involving l.s. planes.
Refinement. Refinement of *F*^2^ against ALL reflections. The weighted *R*-factor *wR* and goodness of fit *S* are based on *F*^2^, conventional *R*-factors *R* are based on *F*, with *F* set to zero for negative *F*^2^. The threshold expression of *F*^2^ > σ(*F*^2^) is used only for calculating *R*-factors(gt) *etc*. and is not relevant to the choice of reflections for refinement. *R*-factors based on *F*^2^ are statistically about twice as large as those based on *F*, and *R*- factors based on ALL data will be even larger.

### Fractional atomic coordinates and isotropic or equivalent isotropic displacement parameters (Å^2^)

**Table:**

	*x*	*y*	*z*	*U*_iso_*/*U*_eq_	
Cl1	0.74850 (9)	−0.11634 (3)	0.17747 (2)	0.02647 (10)	
O1	1.1404 (3)	−0.24034 (9)	−0.00816 (7)	0.0278 (2)	
O2	0.6357 (3)	0.14675 (10)	0.16065 (7)	0.0294 (2)	
N1	0.4384 (3)	0.38133 (11)	0.18840 (9)	0.0268 (2)	
N2	0.2005 (4)	0.63890 (12)	0.52232 (10)	0.0347 (3)	
N3	−0.2867 (4)	0.85277 (13)	0.41743 (11)	0.0400 (3)	
C1	1.0708 (3)	−0.12968 (12)	−0.00163 (9)	0.0213 (2)	
C2	0.8789 (3)	−0.04984 (12)	0.08193 (9)	0.0208 (2)	
C3	0.8076 (3)	0.07272 (12)	0.08605 (9)	0.0215 (2)	
C4	0.3922 (4)	0.43073 (12)	0.28796 (10)	0.0254 (3)	
H4	0.4423	0.3805	0.3335	0.031*	
C5	0.2725 (3)	0.55390 (12)	0.32762 (10)	0.0233 (3)	
C6	0.1973 (4)	0.62808 (13)	0.26185 (10)	0.0265 (3)	
H6	0.1169	0.7124	0.2871	0.032*	
C7	0.2431 (4)	0.57525 (13)	0.15812 (11)	0.0281 (3)	
H7	0.1922	0.6227	0.1106	0.034*	
C8	0.3640 (4)	0.45244 (13)	0.12482 (10)	0.0270 (3)	
H8	0.3956	0.4171	0.0537	0.032*	
C9	0.2324 (4)	0.60218 (13)	0.43620 (10)	0.0263 (3)	
C10	−0.2753 (4)	0.87366 (14)	0.50394 (12)	0.0310 (3)	
C11	−0.2586 (4)	0.90013 (16)	0.61421 (12)	0.0365 (3)	
H11A	−0.0933	0.8436	0.6332	0.055*	
H11B	−0.4846	0.8857	0.6406	0.055*	
H11C	−0.1869	0.9889	0.6432	0.055*	
H2	0.596 (7)	0.226 (3)	0.152 (2)	0.082 (9)*	

### Atomic displacement parameters (Å^2^)

**Table:**

	*U*^11^	*U*^22^	*U*^33^	*U*^12^	*U*^13^	*U*^23^
Cl1	0.03569 (18)	0.02348 (16)	0.02309 (16)	0.00415 (12)	0.00557 (12)	0.01100 (11)
O1	0.0404 (5)	0.0181 (4)	0.0255 (5)	0.0062 (4)	0.0045 (4)	0.0065 (4)
O2	0.0445 (6)	0.0199 (5)	0.0242 (5)	0.0089 (4)	0.0100 (4)	0.0058 (4)
N1	0.0349 (6)	0.0203 (5)	0.0252 (5)	0.0066 (4)	0.0041 (5)	0.0061 (4)
N2	0.0488 (8)	0.0284 (6)	0.0272 (6)	0.0078 (5)	0.0018 (5)	0.0075 (5)
N3	0.0512 (8)	0.0302 (7)	0.0380 (7)	0.0070 (6)	0.0065 (6)	0.0076 (5)
C1	0.0267 (6)	0.0180 (5)	0.0194 (5)	0.0010 (4)	−0.0019 (5)	0.0053 (4)
C2	0.0272 (6)	0.0186 (6)	0.0174 (5)	0.0009 (4)	0.0006 (5)	0.0060 (4)
C3	0.0268 (6)	0.0188 (6)	0.0188 (5)	0.0017 (4)	−0.0010 (5)	0.0045 (4)
C4	0.0321 (7)	0.0199 (6)	0.0254 (6)	0.0057 (5)	0.0015 (5)	0.0077 (5)
C5	0.0272 (6)	0.0199 (6)	0.0226 (6)	0.0024 (5)	0.0009 (5)	0.0049 (5)
C6	0.0327 (7)	0.0188 (6)	0.0286 (7)	0.0065 (5)	0.0026 (5)	0.0070 (5)
C7	0.0365 (7)	0.0238 (6)	0.0261 (6)	0.0066 (5)	0.0010 (5)	0.0100 (5)
C8	0.0348 (7)	0.0229 (6)	0.0235 (6)	0.0048 (5)	0.0036 (5)	0.0062 (5)
C9	0.0316 (7)	0.0205 (6)	0.0273 (6)	0.0052 (5)	0.0015 (5)	0.0067 (5)
C10	0.0307 (7)	0.0227 (6)	0.0387 (8)	0.0033 (5)	0.0063 (6)	0.0061 (5)
C11	0.0407 (8)	0.0335 (8)	0.0329 (8)	0.0040 (6)	0.0042 (6)	0.0044 (6)

### Geometric parameters (Å, °)

**Table:**

Cl1—C2	1.7200 (13)	C4—H4	0.9500
O1—C1	1.2201 (15)	C5—C6	1.3884 (18)
O2—C3	1.3099 (15)	C5—C9	1.4399 (18)
O2—H2	0.91 (3)	C6—C7	1.3883 (19)
N1—C4	1.3316 (17)	C6—H6	0.9500
N1—C8	1.3387 (18)	C7—C8	1.3848 (19)
N2—C9	1.1406 (18)	C7—H7	0.9500
N3—C10	1.138 (2)	C8—H8	0.9500
C1—C2	1.4521 (17)	C10—C11	1.451 (2)
C1—C3^i^	1.5153 (18)	C11—H11A	0.9800
C2—C3	1.3546 (17)	C11—H11B	0.9800
C3—C1^i^	1.5153 (18)	C11—H11C	0.9800
C4—C5	1.3955 (18)		
			
C3—O2—H2	114.0 (17)	C7—C6—C5	117.96 (12)
C4—N1—C8	118.49 (11)	C7—C6—H6	121.0
O1—C1—C2	123.93 (12)	C5—C6—H6	121.0
O1—C1—C3^i^	117.75 (11)	C8—C7—C6	119.07 (12)
C2—C1—C3^i^	118.31 (10)	C8—C7—H7	120.5
C3—C2—C1	121.90 (11)	C6—C7—H7	120.5
C3—C2—Cl1	120.77 (10)	N1—C8—C7	122.88 (13)
C1—C2—Cl1	117.33 (9)	N1—C8—H8	118.6
O2—C3—C2	123.08 (12)	C7—C8—H8	118.6
O2—C3—C1^i^	117.14 (11)	N2—C9—C5	179.14 (15)
C2—C3—C1^i^	119.78 (11)	N3—C10—C11	179.69 (18)
N1—C4—C5	122.13 (12)	C10—C11—H11A	109.5
N1—C4—H4	118.9	C10—C11—H11B	109.5
C5—C4—H4	118.9	H11A—C11—H11B	109.5
C6—C5—C4	119.46 (12)	C10—C11—H11C	109.5
C6—C5—C9	121.20 (12)	H11A—C11—H11C	109.5
C4—C5—C9	119.34 (12)	H11B—C11—H11C	109.5
			
O1—C1—C2—C3	179.96 (13)	C8—N1—C4—C5	−0.6 (2)
C3^i^—C1—C2—C3	−0.1 (2)	N1—C4—C5—C6	0.2 (2)
O1—C1—C2—Cl1	0.78 (18)	N1—C4—C5—C9	−179.19 (13)
C3^i^—C1—C2—Cl1	−179.23 (9)	C4—C5—C6—C7	0.4 (2)
C1—C2—C3—O2	−179.86 (12)	C9—C5—C6—C7	179.82 (13)
Cl1—C2—C3—O2	−0.72 (19)	C5—C6—C7—C8	−0.6 (2)
C1—C2—C3—C1^i^	0.1 (2)	C4—N1—C8—C7	0.4 (2)
Cl1—C2—C3—C1^i^	179.21 (9)	C6—C7—C8—N1	0.3 (2)

Symmetry codes: (i) −*x*+2, −*y*, −*z*.

### Hydrogen-bond geometry (Å, °)

**Table:**

*D*—H···*A*	*D*—H	H···*A*	*D*···*A*	*D*—H···*A*
O2—H2···N1	0.92 (3)	1.75 (3)	2.6111 (17)	154 (3)
O2—H2···O1^i^	0.92 (3)	2.25 (3)	2.6824 (14)	108 (2)
C4—H4···N2^ii^	0.95	2.46	3.292 (2)	146
C6—H6···N3	0.95	2.57	3.385 (2)	144
C7—H7···O1^iii^	0.95	2.48	3.4248 (18)	172
C11—H11A···N2	0.98	2.62	3.341 (2)	130

Symmetry codes: (i) −*x*+2, −*y*, −*z*; (ii) −*x*+1, −*y*+1, −*z*+1; (iii) *x*−1, *y*+1, *z*.
